# Delivery of Topical Drugs to the Olfactory Cleft

**DOI:** 10.3390/jcm12237387

**Published:** 2023-11-29

**Authors:** Andreas Espehana, Liam Lee, Elizabeth Mairenn Garden, Gabija Klyvyte, Shyam Gokani, Lavandan Jegatheeswaran, Jeremy Jonathan Wong, Carl Philpott

**Affiliations:** 1 Norwich Medical School, University of East Anglia, Norwich NR4 7TJ, UK; liam.lee@uea.ac.uk (L.L.); mairenn.garden@nhs.net (E.M.G.); g.klyvyte@uea.ac.uk (G.K.); shyamg@live.co.uk (S.G.); c.philpott@uea.ac.uk (C.P.); 2 Ear, Nose and Throat (ENT) Department, James Paget University Hospital, James Paget University Hospitals NHS Foundation Trust, Great Yarmouth NR31 6LA, UK; lavandan.jegatheeswaran@nhs.net; 3 Ear, Nose and Throat (ENT) Department, Norfolk and Norwich University Hospital, Colney Lane, Norwich NR4 7UY, UK; jeremy.wong3@nhs.net

**Keywords:** olfactory dysfunction, chronic rhinosinusitis, olfaction

## Abstract

Olfactory dysfunction affects approximately 20% of the population globally, with incidence increasing over the age of 60. The pathophysiology is complex, not yet fully understood, and depends on many factors, including the underlying cause. Despite this, the present literature on olfaction is limited due to significant heterogeneity in methodological approaches. This has resulted in limited effective treatments available for olfactory dysfunction. Medications for olfactory dysfunction can be administered locally (directly to the olfactory epithelium) or systemically (orally or intravenously). Currently, there are various methods for local drug delivery to the olfactory epithelium (nasal drops, nasal sprays, atomisers, pressured meter-dosed inhalers, rinses, and exhalation delivery systems). The aims of this review are to summarise the different methods of drug delivery to the olfactory cleft, evaluate the current literature to assess which method is the most effective in delivering drugs to the olfactory epithelium, and review the medications currently available to treat olfactory dysfunction topically. Going forward, further research is required to better establish effective methods of drug delivery to the olfactory epithelium to treat smell disorders.

## 1. Introduction

Since the COVID-19 pandemic, there has been increasing public and scientific interest in the pathophysiology and treatment of olfactory dysfunction. Its impacts are far-reaching, with patients reporting higher rates of depression (49%), anxiety (47%), impairment of eating (95%), isolation (64%), and relationship difficulties (59%) [1]. Olfactory dysfunction can be split into quantitative and qualitative disorders. In the former, a change in the strength of the odour is reported, whilst, in the latter, an alteration in the quality of the odour is noticed.

Estimates of the prevalence of olfactory dysfunction vary between 1.4 and 23% [2], with a meta-analysis of 175,073 patients reporting an overall prevalence of 22.2% [3]. A reason for the variation in prevalence figures reported is thought to be due to the assessment method used, with psychophysical assessment methods yielding a higher prevalence estimate than self-reporting from patients.

An important cause of olfactory dysfunction is inflammatory sinonasal disease, such as chronic rhinosinusitis (CRS), which is estimated to affect 5–12% of the global population. Olfactory dysfunction is a major symptom of CRS, with up to 80% of CRS patients experiencing a reduction or loss of smell, which significantly affects their quality of life. Other important causes of olfactory dysfunction include infectious agents (most notably COVID-19), head trauma, neurological disease, iatrogenic, and congenital, to name a few [2].

There is currently no standardised practice in treating olfactory dysfunction in clinical practice, as the literature on olfactory dysfunction is limited due to significant methodological heterogeneity [2]. As such, an effective treatment for patients, with olfactory dysfunction remains elusive with several therapeutic options being investigated. Currently, corticosteroids are commonly prescribed to treat olfactory dysfunction either orally or topically (via sprays or drops). Newer medications such as Vitamin A and Calcium buffers are also undergoing investigation. However, meaningful evidence of their effectiveness has yet to be found.

Medications for olfactory dysfunction can be administered either locally (directly to the olfactory epithelium) or systemically (orally or intravenously). This review aims to summarise the different methods of drug delivery to the olfactory cleft, look through the current literature to assess which one is most effective, and summarise the various drugs currently being used to treat olfactory dysfunction.

## 2. Nasal Anatomy

The nose is a complex structure with olfactory and respiratory functions. It is divided into two nasal cavities by the septum, and this structure is kept intact by a mixture of bone and cartilaginous framework. Within each cavity, the nose is divided into different regions: the nasal vestibule, the respiratory region, and the olfactory region. Surrounding the nasal cavities are air-filled mucosal-lined sinuses, including the frontal, sphenoid, ethmoid, and maxillary sinuses. Apart from the sphenoid sinuses, all these sinuses communicate with the nasal cavity via ducts that drain through the ostia located on the lateral wall. The sphenoid sinuses empty into the posterior roof of the nasal cavity.

The respiratory region of the nose covers most of the nasal cavity and consists of a mixture of respiratory epithelium and mucous cells with the function of humidifying, warming, protecting, filtering, and eliminating debris from the air that is breathed in. Consequently, the nose naturally filters medications delivered intranasally [4].

Within the olfactory region, odorants are transported to the olfactory epithelium located at the superior apex of the nasal cavity. Mucus helps trap the odorants, which then bind to odorant-binding proteins that concentrate and solubilise these particles. These particles then attach to the olfactory receptors on cilia that transmit signals through the cribriform plate to synapse with the neurons of the olfactory bulb. This, in turn, then sends signals to the olfactory nerve into secondary neurons for higher processing before entering the brain. The apex location of the olfactory cleft within the nasal cavity anatomically may make it difficult for topical drugs to reach the olfactory epithelium [5].

## 3. Different Methods of Topical Administration

Topical drug delivery devices can be categorised into “high volume” devices that deliver at least 50 mL into the nostrils (e.g., squeeze bottles and squirt system) and “low volume” devices, such as nasal sprays or nasal drops.

When delivering medications topically for olfactory dysfunction, the medications need to reach the olfactory epithelium in the olfactory cleft. This is challenging, as the olfactory epithelium is only a few millimetres wide and approximately 7 cm away from the nasal vestibule on average [6]. Along the pathway from the vestibule to the olfactory epithelium lie several intranasal structures that can obstruct medication delivery. To treat olfactory loss in CRS, topical medications applied need to directly target the olfactory cleft, thus improving intrinsic olfactory function. The medications also need to reduce sinonasal tissue inflammation, thus alleviating obstructive olfactory dysfunction and increasing the delivery of other medications directly to the olfactory cleft. Thus, the main challenge is ensuring that the delivery method can deliver medication to this small area to maximise its therapeutic effect.

Several established methods of administering medication to olfactory cleft have been developed, such as nasal sprays, nasal drops using various sitting/lying positions, rinses, atomisers, and directly applying medication with endoscopic guidance.

### 3.1. Nasal Sprays

Nasal sprays have been used for many decades and are the most common way of administering drugs to the olfactory epithelium. They function by aerosolising the drug and transporting it to the nasal epithelium. The most common types of spray pumps are pressurised metred-dose (pMDIs) or pressurised aerosols, single- and duo-dose spray devices. They both deliver similar doses of medication per spray—around 100 mL (range 25–200 mL) per spray.

The characteristics of a device/spray that contains the drug have significant implications in terms of effects, as droplet size and spray angle play a significant role in effective delivery [7]. For example, larger droplet size and a wider spray angle increase the deposition in the nasal vestibule. Current data on the effect of positioning, sniffing, inhaling, or blowing prior to drug administration on the olfactory cleft are not conclusive. Benninger et al. conducted a systematic review to create guidance for patients to optimise drug delivery of intranasal corticosteroid sprays for allergic and nonallergic rhinitis [8]. These guidelines include holding the head in the neutral position, clearing the nose of any mucus, inserting the nozzle into the nostril, and spraying laterally—away from the septum to avoid the potential for epistaxis. Following application, the authors recommend gently inhaling and breathing out through the nose to maximise delivery [8].

However, no such guidance exists for the use of intranasal sprays for olfactory dysfunction. Previous studies demonstrated that most of the liquid delivered using intranasal sprays only reaches the ventral part of the nasal cavity, the largest portion being deposited on the anterior surface of the inferior turbinate [9,10,11], limiting its therapeutic effect. Heilmnann et al. found that two-thirds of patients being treated with local application of corticosteroids via nasal sprays experienced little to no improvement in olfactory dysfunction compared to systemic corticosteroids [12]. They suggested that it could be due to the drug not effectively reaching the olfactory cleft or that local steroids are not as effective as systemic steroids.

Compared to other methods of intranasal drug delivery, nasal sprays are easy to use and have a reduced cost. However, the ability of nasal sprays to reach proximal areas of the sinonasal mucosa is limited, such as sinus cavities in CRS patients after endoscopic sinus surgery (ESS). Muenkaew et al. conducted a randomised trial comparing sinonasal corticosteroid distribution using nasal sprays and nasal irrigations in 40 CRS patients. They found that nasal sprays were inferior to irrigations in reaching the maxillary and anterior ethmoid sinuses when analysed with fluorescein dye during ESS. Both methods had limited fluorescein staining of the frontal and sphenoid sinuses [13].

### 3.2. Nasal Drops with Various Head Positions

**Figure 1:**
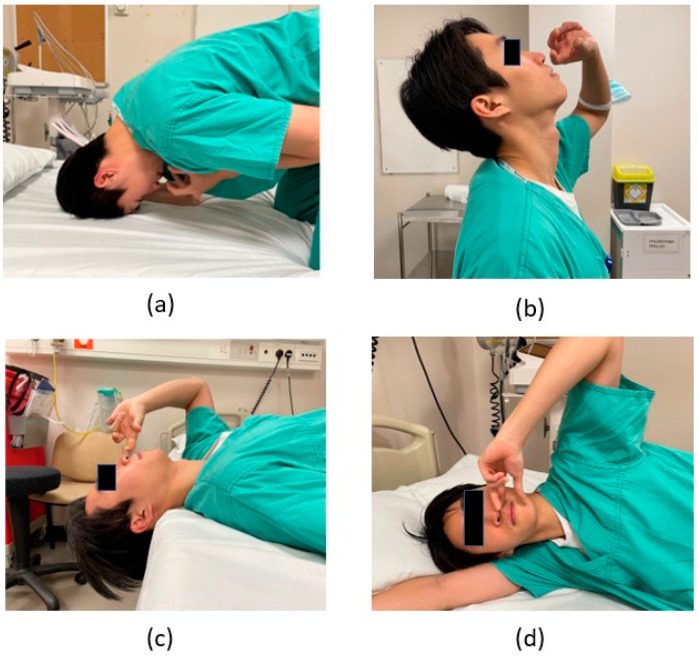
Various positions to apply medication into the nose. (**a**) Head down and forward position (HDF); (**b**) Head back position (HBP); (**c**) Lying head back (LHB); (**d**) Kateiki.

Nasal drops are another common method of delivering topical drugs intranasally, utilising various positions and gravity to transport liquid to the olfactory epithelium. There are several different head positions to improve the use of nasal drops, such as the head back position (HBP), lying head back position (LHB or Mygind), the head down and forward position (HDF), and the Kateiki position. These positions have been demonstrated in Figure 1. However, some of these head positions may be uncomfortable for the patient, which may decrease compliance [14,15] and the effectiveness of the drug.

Of the mentioned head positions, the HBP is the simplest to perform but has been shown to have a nasal drop distribution primarily to the nasal floor, reducing its effectiveness. The LHB and HDF positions have better distribution, with medication reaching the middle meatus and sphenopalatine area [15,16,17]. The Kateiki position is a method of intranasal steroids that Japanese researchers first described in research with cadaveric heads, with Kateiki meaning ‘comfortable’ [16,17,18]. In this position, subjects lie on their sides with their heads tilted and chins turned upwards—a link to a video demonstrating how to perform the Kateiki position can be found in the Supplementary Material. It has been shown in research conducted that this comfortable position can effectively deliver topical treatments to the olfactory epithelium [18]. Mori et al. found that nasal drops reached the olfactory cleft in 96% of the decongested cases and 75% of the cases that had not been decongested. They hypothesised that this might indicate more effective therapies using these methods [18]. Milk et al. compared the LHB position and the Kateiki position, showing that both positions were effective in delivering nasal drops onto the olfactory cleft [19].

When deciding which position to use for the administration of nasal drops, it is important to consider patient preference to ensure compliance.

### 3.3. Rinses/Irrigation

Nasal irrigation/rinses are delivery methods of drugs dissolved in either hypotonic, isotonic, or hypertonic solution into the nose using plastic bottles or syringes. Commonly available products for nasal irrigation include the Neilmed^®^ Sinus Rinse™ system.

No studies have been conducted on analysing the efficacy of irrigation systems in delivering medication specifically to the olfactory cleft. Previous studies suggested that corticosteroid nasal irrigation does not increase the quality of life or grant additional benefits when compared to nasal saline irrigation. These studies were limited by design, heterogeneity of surgical practice, short follow-up, and underpowered analyses [20,21]. Harvey et al. found that in diffuse or patchy chronic rhinosinusitis, the use of nasal irrigation compared with simple nasal spray had greater improvement in nasal blockage, less inflammation, lower overall symptoms on questionnaires, and greater radiological disease suppression. Furthermore, no patients reported medication-associated reactions [22]. Other studies did not report any symptoms of adrenal suppression after 6 months of corticosteroid nasal irrigation unless used with other corticosteroid preparations, such as pulmonary inhalers [23].

Therefore, high-volume nasal irrigation appears to improve nasal symptoms when compared to conventional nasal sprays. However, they do pose a potential risk of adrenal axis suppression in at-risk patient populations. In addition, studies have shown that patients receiving corticosteroids via nasal saline irrigation post-ESS experienced facial pain/pressure, delayed nasal drainage, and ear symptoms.

### 3.4. Atomisers

Intranasal Mucosal Atomisation Devices (MAD) atomise medications into particles with a size as small as 30 to 100 μm, increasing the surface area for absorption [24].

There is conflicting evidence on the effectiveness of atomisers in comparison with nasal drops. A study reported that using the MAD in the LHB position is more effective in delivering medication all around the sinuses and the olfactory cleft compared with the HDF position [25]. However, Cannaday et al. showed that using nasal drops in the Vertex to Floor (VF) position was more effective in delivering medication to the olfactory cleft than the atomiser (Wolf-Tory MADomizer) [26].

### 3.5. The Exhalation Delivery System (OptiNose)

This is a new drug delivery technology that uses exhaled breath to deliver powder or liquid medications. The device prevents potential lung toxicity by avoiding lung deposition. It consists of a mouthpiece and a sealing nosepiece to be placed into a single nostril. The user then exhales through the mouthpiece, which elevates the soft palate and transfers pressure from the mouth to the nose. The air travelling from the mouth enters the device via the mouthpiece, and this way, the powder or liquid can travel around the nasal cavity. The positive pressure created in the nose by the OptiNose device expands the nose that is tightened by inflammation versus sniffing, which is a negative pressure system [27,28].

The OptiNose device has been shown to deposit drugs deeper, more broadly, and posteriorly where polyps originate, with less drip out when compared to conventional sprays [29]. Multiple studies have shown that steroids delivered via OptiNose were an effective way of treating nasal polyps and improved nasal blockage, discomfort, rhinitis, and sense of smell [30,31,32]. However, these studies did not investigate delivery to the olfactory cleft specifically.

### 3.6. Direct Administration under Endoscopic Guidance

Topical medications can be applied directly to the olfactory epithelium using endoscopic guidance. Medications such as platelet-rich plasma (PRP) can be injected directly into the olfactory epithelium or impregnated into dissolvable matrices/sponges such as Gelfoam [33].

The benefits of adopting such a method are that the clinician can ensure the therapeutic medication will interact directly with the olfactory epithelium. However, this requires patients to attend hospital appointments for an experienced clinician to apply this into their nose. Therefore, it may not be suitable for medications that require frequent/daily applications to exert the desired therapeutic effect for olfactory dysfunction.

## 4. Different Topical Agents

### 4.1. Intranasal Corticosteroids

The use of intranasal corticosteroids (such as mometasone and fluticasone) is common in the treatment of olfactory disorders, irrespective of the cause [2]. As in cases of sinonasal inflammatory disease, the treatment aims to reduce inflammation in the olfactory epithelium and to increase olfactory function. Out of all the drugs available to treat olfactory loss, corticosteroids remain the most studied. Corticosteroids can be delivered intranasally via sprays/aerosols, drops, and irrigation.

A large systematic review of 18 randomised controlled trials and 2738 participants found moderate quality evidence in the use of intranasal corticosteroids in the improvement of smell loss in cases of CRS [34]. They found that there is insufficient evidence to suggest which type or method (spray or aerosol) of intranasal steroid should be used in patients with CRS.

Similarly, a meta-analysis found that topical steroids improved the olfactory score in COVID-19 patients at 4 weeks post-treatment (SMD = 1.0440 [0.6777, 1.4102], *p* < 0.0001). However, there was no significant difference in the incidence of full recovery between groups (odds ratio [OR] = 1.4345 [0.9525, 2.1604], *p* = 0.0842) [35]. This supports an international expert review that indicates that there is no long-term benefit of the use of intranasal steroids in post-COVID olfactory dysfunction [36].

For olfactory dysfunction not secondary to sinonasal disease, the evidence for the use of intranasal corticosteroids is limited [2]. Whilst some studies have demonstrated an improvement in olfaction following intranasal steroids [37], the lack of subgroup analysis by aetiology in these studies makes it difficult to draw definitive conclusions [2]. There is a need for larger numbers of high-quality randomised controlled trials to assess their effectiveness in an aetiology-specific manner.

### 4.2. Intranasal Insulin

It is known that insulin receptors can be found in significant amounts in the olfactory epithelium and parts of the central nervous system involved in olfactory processing, such as the hypothalamus, olfactory bulb, and hippocampus. Thus, delivering insulin intranasally to the olfactory epithelium could potentially alter the olfactory and the central processing of olfaction [38]. Intranasal insulin is safe to administer, and a systematic review found that no adverse effects or hypoglycaemia occurred when Intranasal insulin of doses between 10 and 160 IU occurred in 1092 participants [39]. Delivery of insulin is performed using nasal sprays.

There have been few studies conducted to assess the effects of intranasal insulin on olfaction, but the results have been conflicting.

A study of the effect of intranasal insulin (intranasal dose of 40 IU) compared to placebo on healthy individuals with normal olfactory function (categorised by the extended version of Sniffin’ stick identification test) showed that olfactory sensitivity was decreased after receiving intranasal insulin compared to placebo, but there was no effect on olfactory discrimination [40]. A follow-up study by the same authors showed that this effect was only seen in women receiving women receiving intranasal insulin. On the other hand, male patients showed no significant difference between insulin and placebo [41].

A single-centre randomised control trial that investigated the effects of intranasal insulin on participants with hyposmia was investigated. Intranasal insulin was delivered as a gel foam containing 40 IU and placed endoscopically; this procedure was performed on participants twice a week for 4 weeks. Results reported that the intervention group had demonstrated improved olfactory sensitivity to butanol [33].

Another single-centre single-blinded randomised trial was conducted to evaluate the effects of an insulin film on patients with post-COVID olfactory loss. This dissolvable film containing insulin was applied directly onto the olfactory cleft using nasal endoscopy. The treatment group receiving insulin film showed significant improvement in sensitivity to butanol, while the placebo group did not. Similarly, the treatment group saw significant improvement in discrimination ability after receiving treatment, and the placebo group did not show any significant improvement [42].

A separate study investigated the effects of intranasal insulin delivered via a precision air pump on male participants only. The study showed that participants receiving insulin had significant improvement in their olfactory threshold compared to their pre-treatment baseline, but there was no significant improvement in discrimination function. The study also found that the effect was dose-dependent, with better improvement seen with higher doses of insulin (100–160 IU) [43].

### 4.3. Intranasal Theophylline

Theophylline is a phosphodiesterase inhibitor that has been reported to improve olfactory function. In patients with anosmia, lower levels of cyclic adenosine monophosphate and cyclic guanosine monophosphate have been noted. Thus, it appears that theophylline can increase these important molecules in nasal secretions, leading to improved olfactory signalling and axonal regeneration [44,45]. In addition, nasal theophylline administration has been found to reduce Interleukin-10 (IL-10) in nasal mucus. IL-10 is an anti-inflammatory cytokine that helps to modulate the inflammatory response. As postviral anosmia is known to be associated with inflammation, it has been hypothesised that altering the IL-10 pathway may help in hyposmia treatment. Intranasal theophylline has been delivered using irrigation and sprays.

A recent trial has found that intranasal theophylline decreases nasal IL-10 in anosmic/hyposmic patients who had elevated IL-10 levels before the trial. This was also associated with improved smell and taste on olfactory testing. However, the study had a high risk of confounders and bias, as the patients who seemed to have increased smell function were also treated with corticosteroids for other comorbidities such as asthma or nasal polyps [46]. Systemic theophylline has a narrow therapeutic window. A recent randomised controlled trial of 400 mg theophylline nasal irrigation versus placebo for COVID-19-related loss of olfaction reported inconclusive results. However, they reported a 16% improvement in olfactory function; hence, larger studies with higher dosages could show different results [45]. Therefore, more evidence is needed on the use of theophylline, focussing on larger trials, longer duration of treatment, and higher doses of the drug.

### 4.4. Intranasal Tetra Sodium Pyrophosphate/Sodium Citrate

Intranasal calcium concentration has been postulated to play an important role in olfactory signalling [47]. Early studies suggested that free calcium in nasal mucosa inhibits olfactory signalling through the desensitisation of cyclic nucleotides (primarily cAMP), which is a key signalling molecule to initiate cation influx for depolarisation [28,48]. In addition, calcium is important in the phosphorylation of adenylyl cyclase, which subsequently reduces cAMP production [29,49]. It should be noted that the role of calcium is not limited to inhibition. Its function is complex, involved in both excitatory and inhibitory signalling [27]. Nonetheless, recent studies have investigated the effect of lowering intranasal calcium concentration on olfactory function. By far, the most prevalent method was to use calcium chelating agents, in particular, sodium citrate. Intranasal sodium pyrophosphate/citrate can be performed using drops and sprays.

Panagiotopoulos et al. (2005) was one of the first to test this hypothesis [50]. The TDI scores of thirty-one volunteers with hyposmia–anosmia due to various causes were evaluated after the administration of topical saline, topical adrenaline, and topical sodium citrate. The TDI scores were significantly improved within less than one hour of administering sodium citrate. The median time of the patients’ subjective sense of smell improvement duration was 3 h. Similarly, Philpott et al. (2017) investigated the effect of a single administration of sodium citrate and its short-term effects (measured up to 2 h), similar to Panagiotopoulos et al. [51]. This study demonstrated a temporary improvement, 30–120 min, in threshold test in postviral hyposmic–anosmic patients treated with sodium citrate compared to the saline group.

Whitcroft et al. [52] conducted a series of prospective placebo-controlled studies of sodium citrate. Specifically, a subgroup analysis showed the improvement in the Sniffin Stick identification score in the postviral anosmia group 20–30 min after treatment. A follow-up study focussed on 49 patients with postviral olfactory impairment. There was a statistically significant improvement in composite threshold + identification score in participants with postviral hyposmia after a one-off application of sodium citrate. Although statistically significant, the increase in threshold and identification was not clinically significant (<5.5 points improvement combined). In addition, there was no statistically significant difference between the sodium citrate group and the sodium chloride (placebo) group in terms of their threshold and identification scores. Contrary to their previous findings, a prolonged application of sodium citrate for 2 weeks showed no clinically significant effect on the composite TDI score or for each individual T, D, and I score. It is important to note that these studies used the contralateral nostril as the control for internal control. However, there is evidence that the olfactory function of nostrils is asymmetrical—a limitation that the study recognised.

Other chelating agents have been trialled in post-COVID-19 anosmic patients by Abdelazim et al., including tetrasodium pyrophosphate, sodium gluconate, and nitrilotriacetic acid. All studies demonstrated a significant decrease in intranasal calcium and improvement in TDI scores 1 month after treatment commencement. However, these studies were poor in quality—lacking adequate detail for exclusion/inclusion criteria for reproducibility, failure to provide a power calculation, and inclusion of participants 14 days post-COVID, which has a high likelihood of spontaneous recovery. Therefore, the findings must be considered with caution [53,54,55].

As such, although chelating agents are mostly well tolerated with few side effects, the current data are not sufficient to implement these agents into routine clinical practice.

### 4.5. Intranasal Platelet-Rich Plasma

A promising feature of olfactory neuroepithelium in mammals is its ability to self-renew as compensation for its short lifespan. Therefore, this sparked the idea of using a topical treatment that may aid the regeneration of damaged olfactory neuroepithelial cells or basal stem cells important in maintaining olfactory neuroepithelial cells. Platelet-rich plasma (PRP) is a relatively novel material that contains growth factors important in tissue repair with a high concentration of platelets and has been used to treat olfactory dysfunction with direct injection to the olfactory cleft.

The possible utility of PRP in olfactory dysfunction was suggested recently in a small preliminary study by Mavrogeni et al. (2017) [56]. In this study, four out of five participants with refractory anosmia reported the restoration of their smell after four topical administrations across 3 months. Another pilot study of seven patients found that three out of five patients with hyposmia achieved normosmia in a 3-month follow-up after a single administration of PRP [57]. The clinical evidence was supported through mice models, which demonstrated improved food-finding time and a thickened olfactory epithelium on histopathology.

PRP has also been explored in the context of post-COVID-19. There was a significant improvement in the visual analogue scale for parosmia in the group treated with PRP compared to the control group, who received a continued prestudy intervention. For post-COVID-19 anosmia, so far, one case has been published, which demonstrated improvement in the sense of smell after 3 weeks postinjection.

Although promising, the evidence remains limited, and the benefits of PRP need to be further supported by larger clinical studies.

### 4.6. Vitamin A

Retinoic acid is a known metabolite of vitamin A; its role as a transcription regulator is crucial to tissue development and regeneration, particularly in olfactory system embryogenesis and adult neuronal regeneration. Hence, the theory is that topical application of Vitamin A to the olfactory epithelium, delivered primarily with intranasal drops, can induce neuronal regeneration, hence improving olfaction [58,59].

Limited studies have been conducted to investigate the effectiveness of vitamin A in the treatment of olfactory loss. The first evidence was a case series by Duncan et al., who reported an improvement in olfaction in patients after being treated with systemic vitamin A [60]. Most recently, Hummel et al. conducted a study comparing the treatment of postviral olfactory loss using smell training versus smell training and vitamin A nasal drops. The study showed that the group receiving vitamin A along with smell training showed greater improvement in smell (assessed using Sniffin’ sticks) compared to just smell training alone [61].

A randomised control trial is being conducted in England to compare the effectiveness of vitamin A in the treatment of postviral olfactory loss [62], but further research is required to determine this.

### 4.7. Omega-3

Omega-3 is a polyunsaturated fatty acid commonly found in fish oil that is responsible for multiple cellular functions. It is also involved in the regulation of the nervous system, blood pressure, clotting, glucose tolerance, and inflammation. Evidence has also demonstrated that taking Omega-3 has benefits for cardiovascular health and improves inflammatory conditions such as asthma and arthritis [63]. So far, it has been used in nasal irrigations.

A study by Greiner et al. has shown that low levels of Docosahexaenoic acid (DHA), a type of omega-3 fatty acid, are related to reduced odour discrimination in rats [64].

A randomised control trial was conducted by Yan et al. using nasal irrigation containing omega-3 versus placebo on patients who underwent resection of sellar or parasellar mass. The study demonstrated that the group receiving omega-3 nasal irrigation was found to have beneficial effects on olfactory loss after controlling for multiple confounding variables [65]. Further studies and research are required to determine their effectiveness in the treatment of olfactory dysfunction.

## 5. Discussion

The delivery of medication to the olfactory epithelium is a critical aspect of the treatment of olfactory disorders. In this regard, the anatomy of the sinonasal space presents a significant challenge in effective drug delivery to the olfactory mucosa. As the middle turbinate obstructs the passage to the olfactory mucosa, most medications applied using conventional methods do not reach the olfactory cleft [6]. Therefore, the therapeutic effectiveness of these medications is greatly reduced.

The application of topical drugs in olfactory dysfunction treatment is a continually evolving field. A summary of medications used is provided in Table 1. Our research contributes to the growing body of literature on various delivery methods such as OptiNose, atomisers, and rinses. However, comprehensive comparative studies examining their efficacy in specifically reaching the olfactory epithelium are still limited. While we have summarised many studies looking into the effectiveness of different delivery methods, comparing across studies is difficult due to the heterogeneity of outcomes measured. Some studies have used clinical outcomes such as symptoms and patient-reported outcome measures (PROMS), while others used more direct measures of the effectiveness of delivery, such as endoscopic evaluation of nasal passageways. Subjective assessment methods, like direct visualisation through nasal endoscopy, can provide initial insights. For example, Scheibe et al. (2008) utilised dye staining to compare the effectiveness of nasal spray, drops, and a squirt system designed by the authors in delivering medication to the olfactory cleft. Their results indicated that the squirt method achieved the highest medication delivery to the olfactory cleft [6].

Further enhancing these subjective approaches, more objective and quantifiable methods are proving to be invaluable. A study by Lam et al. (2013) [66] employed image-based pixel analysis on eight cadaver heads to compare the efficacy of nasal irrigations with pressurised sprays. These objective measures offer a more robust comparison of delivery methods. However, the transition from cadaveric models to living human physiology introduces additional complexities. Therefore, we align with Hura et al. (2020) [67] in advocating for more comprehensive investigations into these novel delivery systems, emphasising the need to confirm these findings in clinical settings.

In terms of the drugs themselves, many have been trialled and studied. However, the number of trials is limited, and those that have been conducted have small participant numbers, which limits the quality of the evidence. There is also great heterogeneity in the methodologies and outcome measures used in the studies assessing the efficacy of drugs in treating olfactory loss. These factors make it difficult to perform high-quality systematic reviews and meta-analyses on this topic.

Recently, a core outcome set (COS) for olfactory disorders was developed by the Clinical Olfactory Working Group (COWoG). The COS was derived from a two-stage Delphi process and aimed to standardise the outcome measures used in trials within a specific area or topic [68]. The COS for olfactory disorders includes a mixture of qualitative and quantitative measures. For the objective measuring of smell and taste, the Sniffin Stick test or the University of Pennsylvania identification test (UPSIT) is recommended [69].

By standardising the outcome measures used in studies, it will be possible to develop meta-analyses on this topic and thus generate higher-quality evidence. Moreover, it is important to note that this paper only discussed drugs that are applied directly to the olfactory epithelium. Medications administered systemically (either orally or intravenously) were not included.

## 6. Conclusions

Our study highlights the complexities in delivering medication to the olfactory epithelium, especially in inflamed sinonasal spaces like in CRS. The potential for topical drug delivery systems in this context is significant, as they offer a more targeted approach, potentially improving treatment efficacy and patient outcomes.

The importance of our findings lies in their implication for future research. We recommend more standardised studies to evaluate and compare different drug delivery systems to the olfactory cleft. Aligning with the recent development of the Core Outcome Set for olfactory disorders, future studies should adopt these standardised measures to ensure consistency and comparability across research.

In summary, our research contributes to a nuanced understanding of the challenges and opportunities in topical drug delivery for olfactory disorders. By exploring and innovating in this field, we can pave the way for more effective treatment strategies, ultimately improving the quality of life for those suffering from olfactory disorders.

## Figures and Tables

**Table 1:** Summary table of different drugs available for topical treatment for smell loss.

Treatment Method	Drug	Mechanism of Action	Clinical Application	Method of Application	References
Intranasal Corticosteroids	Anti-inflammatory agents	Reduces inflammation in the olfactory epithelium	Chronic rhinosinusitis	Sprays/aerosols Drops Irrigations	[2,23,24]
Intranasal Insulin	Hormone involved in glucose metabolism and cell growth	Stimulates olfactory stem cell proliferation and differentiation	Postviral olfactory dysfunction, general olfactory sensitivity	Sprays Gelfoam	[39,40]
Intranasal Theophylline	Phosphodiesterase inhibitor	Increases cyclic adenosine monophosphate and cyclic guanosine monophosphate levels in nasal secretions	General olfactory sensitivity	Irrigation sprays	[45,46]
Intranasal Tetra Sodium Pyrophosphate/Sodium Citrate	Calcium chelating agent	Lowers intranasal calcium concentration to improve olfactory signalling	Postviral olfactory dysfunction, general olfactory sensitivity	Drops Sprays	[28,47,50,51]
Intranasal Platelet-Rich Plasma	Contains growth factors important in tissue repair	Aids regeneration of damaged olfactory neuroepithelial cells or basal stem cells	Postviral olfactory dysfunction, general olfactory sensitivity	Injection	[56,57]
Vitamin A	Metabolite of vitamin A	Induces neuronal regeneration in the olfactory epithelium	Postviral olfactory dysfunction, general olfactory sensitivity	Drops	[58,59,60,61]
Omega-3	Polyunsaturated fatty acid	Enhances membrane fluidity and synaptic function	General olfactory sensitivity	Irrigation	[63,64,65]

## Data Availability

Not applicable.

## Supplementary Materials

The following supporting information can be downloaded at https://www.youtube.com/watch?v=R1z7sy0MPlc&t=2s&ab_channel=FifthSense, Video S1: Apollo Trial Video, How to apply nasal drops.
